# Knowledge of antibiotics and antibiotic resistance among Norwegian pharmacy customers – a cross-sectional study

**DOI:** 10.1186/s12889-019-6409-x

**Published:** 2019-01-15

**Authors:** Marit Waaseth, Abdifatah Adan, Ingrid L. Røen, Karoline Eriksen, Tijana Stanojevic, Kjell H. Halvorsen, Beate H. Garcia, Lone Holst, Karen M. Ulshagen, Hege S. Blix, Hilde Ariansen, Hedvig M. E. Nordeng

**Affiliations:** 1 Department of Pharmacy, UiT The Arctic University of Norway, PO Box 6050 Langnes, N-9037 Tromsø, Norway; 2 Department of Global Public Health and Primary Care, University of Bergen, PO Box 7804, N-5020 Bergen, Norway; 3 Pharmacoepidemiology and Drug Safety Research Group, School of Pharmacy, University of Oslo, PO Box 1068 Blindern, N-0316 Oslo, Norway; 4 Norwegian Medicines Agency, PO Box 6167 Etterstad, N-0602 Oslo, Norway; 5 Division of Mental and Physical Health, Norwegian Institute of Public Health, PO Box 4404 Nydalen, N-0403 Oslo, Norway

**Keywords:** Antibiotics, Antibiotic resistance, Antibiotic knowledge, Attitudes, Beliefs about medicines, BMQ

## Abstract

**Background:**

Antibiotic resistance is a global health threat. Public knowledge is considered a prerequisite for appropriate use of antibiotics and limited spread of antibiotic resistance. Our aim was to examine the level of knowledge of antibiotics and antibiotic resistance among Norwegian pharmacy customers, and to assess to which degree beliefs, attitudes and sociodemographic factors are associated with this knowledge.

**Methods:**

A questionnaire based, cross-sectional study was conducted among pharmacy customers in three Norwegian cities. The questionnaire covered 1) knowledge of antibiotics (13 statements) and antibiotic resistance (10 statements), 2) the general beliefs about medicines questionnaire (BMQ *general)* (three subdomains, four statements each), 3) attitudes toward antibiotic use (four statements), and 4) sociodemographic factors, life style and health. High knowledge level was defined as > 66% of maximum score. Factors associated with knowledge of antibiotics and antibiotic resistance were investigated through univariate and multiple linear regression. Hierarchical model regression was used to estimate a population average knowledge score weighted for age, gender and level of education.

**Results:**

Among 877 participants, 57% had high knowledge of antibiotics in general and 71% had high knowledge of antibiotic resistance. More than 90% knew that bacteria can become resistant against antibiotics and that unnecessary use of antibiotics can make them less effective. Simultaneously, more than 30% erroneously stated that antibiotics are effective against viruses, colds or influenza. Factors positively associated with antibiotic knowledge were health professional background, high education level, and a positive view on the value of medications in general. Male gender, a less restrictive attitude toward antibiotic use, and young age were negatively associated with antibiotic knowledge. The mean overall antibiotic knowledge score was relatively high (15.6 out of maximum 23 with estimated weighted population score at 14.8).

**Conclusions:**

Despite a high level of knowledge of antibiotics and antibiotic resistance among Norwegian pharmacy customers, there are obvious knowledge gaps. We suggest that action is taken to increase the knowledge level, and particularly target people in vocational, male dominated occupations outside the health service, and primary/secondary school curricula.

**Electronic supplementary material:**

The online version of this article (10.1186/s12889-019-6409-x) contains supplementary material, which is available to authorized users.

## Background

According to the World Health Organization (WHO), *“Antibiotic resistance is one of the biggest threats to global health, food security, and development today”* [1]. Increased use of antibiotics worldwide, both in human medicine and in agriculture, has led to increased occurrence of resistant bacteria [2]. Parallel to the spread of bacterial resistance, there has been a decline in research and development of new antibiotics [3].

Inappropriate prescribing and/or overuse of antibiotics among patients are regarded among the main causes of the emergence of resistant bacteria [3]. Although Norway has relatively low use of antibiotics (18 DDD/1000 inhabitants/day in 2016) and can show a reduction in overall consumption in later years [4, 5], there are some causes for concern. The proportion of broad-spectrum antibiotic use has increased [5]. Also, we have seen an increased number of carriers, but also infections, of Methicillin-resistant *Staphylococcus aureus* (MRSA) since 2010, in addition to several major outbreaks of Vancomycin-resistant enterococci (VRE) [6].

The Norwegian national strategy against antibiotic resistance (2015–2020) aims to reduce antibiotic use (measured in DDD/1000 inhabitants/day) by 30% from 2012 to 2020 [7], i.e. from 21 to 15 DDD/1000 inhabitants/day [5]. One of the means to achieve this aim has been to shorten the period of validity for antibiotic prescriptions from one year to ten days, effective from January 2018. Other actions include information campaigns toward both health professions and the general population.

The European strategy describes guidelines for the member states [8]. It does not specify a definite aim for reduced use of antibiotics, but states that the EU will monitor the progress through key outcome indicators. Several actions are planned, e.g. to support national awareness-raising efforts through *“communication tools targeting key audiences and contribute to the annual European Antibiotic Awareness Day (EAAD)”* [8].

Independent information and public education about medicines are two of the 12 key interventions that WHO advocates to promote more rational medicine use in general [9]. When it comes to antibiotics, the first strategic objective of the WHO Global action plan on antimicrobial resistance is to improve public awareness and understanding of the problem [10]. The Norwegian and European strategies likewise emphasizes increased public knowledge [7, 8]. A European survey (excl. Norway), the 2016 Eurobarometer, shows great variability between countries regarding public knowledge of antibiotics [11].

Information campaigns have been launched in several countries, with varying effects on antibiotic use [12]. As a part of the national strategy to increase public knowledge, the Norwegian Ministry of Health and Care Services launched a public information campaign, conducted by the Norwegian Directorate of Health in 2016, about the consequences of antibiotic resistance [13]. The campaign were primarily aimed at social media (Facebook) and included two short videos as well as magazine advertisements and boards produced by a communication agency. The target population was mothers with children aged < 6 years and adults 25–44 years although the posts were open for all [14]. By October 2018, the two videos had reached 789 k and 195 k viewings respectively (https://www.facebook.com/helsedirektoratet/videos/1282170395192759/ and https://www.facebook.com/helsedirektoratet/videos/1249111068498692/).

Independently of official information strategies, media may have covered various aspects of the antibiotic resistance challenge [15, 16]. In Norway, in addition to numerous newspaper articles, a reputable documentary series on the main national broadcasting channel addressed antibiotic resistance in March 2016 [17]. Social media have become important information channels, but may not reach people with low knowledge and/or low interest in the subject [18, 19].

Awareness about antibiotic resistance has been shown to be lower in countries where antibiotic resistance is high [20]. Within the EU, countries with low use of antibiotics, such as Sweden and The Netherlands, show a higher population knowledge level [11]. Consequently, the hypothesis is that increased knowledge of antibiotics among the general population can help achieve appropriate use, better treatment adherence and results, and consequently reduce bacterial resistance. People’s attitudes, beliefs and views regarding medicines may influence their knowledge of antibiotics and antibiotic resistance [20–23]. So far, this has not been investigated in a Norwegian population.

## Aim

The primary aim was to examine the level of knowledge of antibiotics and antibiotic resistance in the Norwegian population, explored among pharmacy customers. The secondary aim was to assess to which degree beliefs, attitudes and sociodemographic factors are associated with this knowledge.

## Material and methods

We carried out a questionnaire based, cross-sectional study among pharmacy customers from 20 pharmacies in three Norwegian cities representing different parts of the country: Tromsø in the north (~ 80,000 inhabitants), Bergen in the southwest (~ 272,000 inhabitants) and Skien in the southeast (~ 55,000 inhabitants)). These cities were selected based on the location of the collaborating university departments represented in the project team, as well as their proximity to the area of residency of the master students performing the data collection. The pharmacies were chosen either based on their large client base (six in Bergen and two in Skien), or all pharmacies in the municipality were chosen (Tromsø, 12 pharmacies). Four pharmacy students (in their final (5th) year) from the Universities of Tromsø, Bergen and Oslo collaborated on the data collection, which took place from October 2016 to February 2017. The inclusion criteria were ≥ 18 years of age and ability to understand Norwegian. The master students were present in the pharmacy at all times during data collection to facilitate questionnaire completion, e.g. show them to the pharmacy information room or provide postage paid envelopes if they were in a hurry, and to prevent misunderstandings if they had difficulties understanding some of the questions. All individuals who entered the pharmacy during the appointed time for data collection were invited to participate.

A structured questionnaire in Norwegian was developed by the project team consisting of researchers with extensive experience in conducting questionnaire-based studies as well as clinical experience and expertise in pharmacy practice. The master students conducted a face validity study for assessment of comprehensibility and completion time among persons without health professional background (*N* = 29). Based on the results from this study, the questionnaire was shortened and some of the questions were rephrased. The revised version, tested among an additional six persons in Tromsø, showed that mean completion time at this study site was reduced from 41 min for the original questionnaire, to 26 min for the revised version. Participants from the face validity study were excluded from the main study. The complete questionnaire was not validated, but included some validated questions, i.e. the Beliefs about Medicines Questionnaire (BMQ) [24]. An English translation of the questionnaire is available upon request.

### Measures

The questionnaire was organised in four parts: 1) knowledge of antibiotics and antibiotic resistance, 2) general beliefs about medicines, 3) attitudes toward antibiotic use and 4) sociodemographic factors, lifestyle and health.

#### Knowledge of antibiotics and antibiotic resistance

The antibiotic knowledge part consisted of 23 statements, 13 regarding antibiotics in general and 10 regarding antibiotic resistance. Six items were sampled from the Eurobarometer [11], and the remaining items were based on experience from clinical or pharmacy settings. Response alternatives were “True”, “False” and “Do not know”. Results are presented as percentage correct answers per statement. For each statement, knowledge was considered satisfactory if ≥80% of the participants answered correctly. Additionally, an overall knowledge score across items was calculated by giving each item one point for correct answer, while wrong answer, “do not know” and missing were set to zero. The overall maximum score was 23, i.e. 13 for antibiotics in general and 10 for antibiotic resistance. High knowledge level was defined as a mean score > 66% of maximum score (the double of what would be achievable on average by random answering), i.e. > 8.66 for antibiotics in general and > 6.66 for antibiotic resistance.

#### General beliefs about medicines

Beliefs about medicines were measured by the beliefs about medicines questionnaire (BMQ *general)* [24]. The instrument consists of three domains: BMQ overuse, BMQ harm and BMQ benefit, each containing four items (belief statements) measured by a 5-point Likert scale; strongly disagree (1), disagree (2), uncertain (3), agree (4) and strongly agree (5). Each domain gives a score from 4 to 20. The Norwegian language version of BMQ overuse and harm has been validated [25], but not BMQ benefit. In addition to total score sum, the mean score per domain was calculated as overall score divided by the number of items per domain, making it comparable to the Likert scale.

#### Attitudes toward antibiotic use

To measure attitudes toward antibiotics, we used four statements from a web survey commissioned by the Norwegian Directorate of Health [26]: “I only wish to use antibiotics if it is necessary”, “The doctor should not give me antibiotics when he/she thinks I do not need it”, “I wish to use antibiotics if I get well sooner”, and “The doctor should give me antibiotics when I think I need it”. We used the same 5-point Likert scale as described above. As a restrictive attitude is preferred when it comes to antibiotic use, we combined the first two statement into a total score (2–10) for restrictive attitude toward antibiotics (i.e. “only if necessary” and “not if I do not need it”). Similarly, the last two statements were combined into a total score for less restrictive attitude (i.e. “if I get well sooner” and “when I think I need it”). As for the BMQ, the mean score per domain was calculated as overall score divided by the number of items per domain, making it comparable to the Likert scale.

#### Sociodemographic factors, life style and health

We included questions on age, gender, work situation, level of education, health professional background, marital status, health status, chronic disease, regular medication use, antibiotic use in the previous 12 months and smoking. Variables were categorized as presented in Table 1. Work situation was categorized as in active work, not working (but of working age, i.e. unemployed, housewife etc.), student and retired. The education categories were primary/lower secondary school, upper secondary school, college/university ≤3 years and college/university > 3 years. We merged the college/university categories when calculating a weighted knowledge score for the general population, because Statistics Norway splits higher education at ≤4 years/> 4 years. Chronic disease was defined as disease that progresses slowly, is long-lasting or recurrent.

**Table 1:** Participants characteristics and proportion with high antibiotic knowledge score^a^

	n	(%)	High antibiotics knowledge score^b^			
			Antibiotics in general		Antibiotic resistance	
			n	(%)	n	(%)
Age						
18–29 years	178	(20.3)	91	(51.1)	104	(58.4)
30–44 years	166	(18.9)	111	(66.9)	123	(74.1)
45–59 years	215	(24.5)	135	(62.8)	163	(75.8)
> =60 years	316	(36.0)	164	(51.9)	233	(73.7)
Gender						
Women	598	(68.2)	384	(64.2)	428	(71.5)
Men	277	(31.6)	117	(42.2)	195	(70.4)
Health statement I have good health						
Strongly agree	240	(27.4)	140	(58.3)	175	(72.9)
Agree	418	(47.7)	244	(58.4)	306	(73.2)
Unsure	102	(11.6)	51	(50.0)	66	(64.7)
Disagree	94	(10.7)	56	(59.6)	64	(68.1)
Strongly disagree	18	(2.1)	9	(50.0)	9	(50.0)
Chronic disease						
Yes	381	(43.4)	256	(67.2)	275	(72.2)
No	470	(53.6)	233	(49.6)	333	(70.9)
Will not report	18	(2.1)	9	(50.0)	10	(55.6)
Smoking						
Yes	90	(10.3)	49	(54.4)	57	(63.3)
No	732	(83.8)	424	(57.9)	535	(73.1)
Sometimes	52	(5.9)	28	(53.8)	31	(59.6)
Regular medication use						
Yes	498	(57.0)	280	(56.2)	355	(71.3)
No	314	(35.9)	186	(59.2)	228	(72.6)
Sometimes	62	(7.1)	34	(54.8)	38	(61.3)
Antibiotics use last 12 months						
Yes	262	(29.9)	164	(62.6)	184	(70.2)
No	602	(68.6)	332	(55.2)	432	(71.8)
Do not remember	12	(1.4)	5	(41.7)	7	(58.3)
Education						
Primary/lower secondary school	69	(7.9)	29	(42.0)	38	(55.1)
Upper secondary school	307	(35.0)	162	(52.8)	190	(61.9)
College/University ≤3 years	242	(27.6)	133	(55.0)	185	(76.4)
College/University > 3 years	247	(28.2)	171	(69.2)	205	(83.0)
Health professional background						
Yes	233	(26.6)	189	(81.1)	196	(84.1)
No	609	(69.4)	291	(47.8)	407	(66.8)
Do not know	17	(1.9))	12	(70.6)	8	(47.1)
Marital status						
Married/cohabiting	546	(62.3)	331	(60.6)	410	(75.1)
In a relationship	71	(8.1)	33	(46.5)	44	(61.9)
Single	232	(26.5)	123	(53.0)	152	(65.5)
Other	23	(2.6)	12	(52.2)	14	(60.9)
Work situation						
In active work	292	(33.3)	190	(65.1)	231	(79.1)
Not working	226	(25.8)	140	(61.9)	148	(65.5)
Student	90	(10.3)	47	(52.2)	54	(60.0)
Retired	249	(28.4)	113	(45.4)	178	(71.5)
Beliefs about medicines^c^						
Beneficial						
Agree	818	(93.3)	472	(57.7)	593	(72.5)
Uncertain	32	(3.6)	12	(37.5)	13	(40.6)
Disagree	27	(3.1)	17	(63.0)	18	(66.7)
Harmful						
Agree	169	(19.3)	82	(48.5)	109	(64.5)
Uncertain	112	(12.8)	65	(58.0)	71	(63.4)
Disagree	596	(68.0)	354	(59.4)	444	(74.5)
Overused						
Agree	465	(53.0)	274	(58.9)	336	(72.3)
Uncertain	154	(17.6)	81	(52.6)	99	(64.3)
Disagree	258	(29.4)	146	(56.6)	189	(73.3)
Attitude toward antibiotics^c^						
Restrictive attitude supported						
Agree	836	(95.3)	483	(57.8)	600	(71.8)
Uncertain	35	(4.0)	16	(45.7)	20	(57.1)
Disagree	5	(0.6)	1	(20.0)	3	(60.0)
Non-restrictive attitude supported						
Agree	215	(24.5)	107	(49.8)	147	(68.4)
Uncertain	185	(21.3)	99	(53.5)	119	(64.3)
Disagree	476	(54.3)	294	(61.8)	357	(75.0)

^a^Proportion with mean score > 66% of maximum score (i.e. > 8.58 on general knowledge and > 6.66 on antibiotic resistance knowledge)

^b^Ten participants had “do not know” on all 23 knowledge items while 59 had missing on at least one item. Number of missing per knowledge item varied from 0 to 11. All missing values were set to zero

^c^Total score transposed to a 1–5 scale (mean of mean) and categorized as agree (4–5), uncertain (3) and disagree (1–2)

### Statistical analyses

We used R version 3.4.1, R Foundation for Statistical Computing, Vienna, Austria (https://www.R-project.org/) for the statistical analyses. We present descriptive statistics as means with standard deviations (SD) or frequencies with proportions (%). Factors associated with knowledge of antibiotics and antibiotic resistance were investigated through univariate and multiple linear regression. Inspection of diagnostic plots of residuals (normal QQ, Residuals vs Fitted, Scale-Location and Residuals vs Leverage) did not reveal serious violation of test assumptions. We performed hierarchical model regression to estimate an average knowledge score for the general population, weighted for age, gender and education.

Statistical significance was defined as *p*-values < 0.001 to adjust for multiple testing

For the BMQ and the attitude scores, participants with missing on more than one item within a subdomain were excluded from the analyses (*n* = 3). For other participants, missing values were imputed by setting missing to 3 on the Likert scale (uncertain) (21 values were imputed, 15 in BMQ and 6 in attitudes).

## Results

The total study population comprised 877 participants out of 2573 invited (overall response rate 34, 19% in Bergen, 55% in Tromsø and 62% in Skien). Mean age was 49.6 (SD 18.4) years. One third of the participants were ≥ 60 years and two thirds were women. Seventy-five percent agreed that they had good health, 56% reported higher education, 43% had a chronic disease, 57% used medication regularly and 30% had used antibiotics during the last 12 months.

### Level of knowledge of antibiotics and antibiotic resistance

Overall, the proportion with high knowledge (> 66% of maximum score) was 57% for antibiotics in general and 71% for antibiotic resistance. Fifty percent had high knowledge level on both domains, while 21% had low knowledge level on both domains.

Table 1 shows the participant characteristics and the corresponding proportion with high level of knowledge. Among participants with a health professional background, more than 80% had high knowledge score on both domains, and people with the highest education showed similar results for antibiotic resistance. Groups with low knowledge level included young people (< 60% with high knowledge), the lowest education category (42 to 55%), men (42% on antibiotics in general), and several relatively small groups of people who expressed variations of uncertainty (e.g. “uncertain” (Likert), “do not know”, “do not remember”, “sometimes”) (from 20% (*n* = 1), to around 60%).

**Fig. 1:**
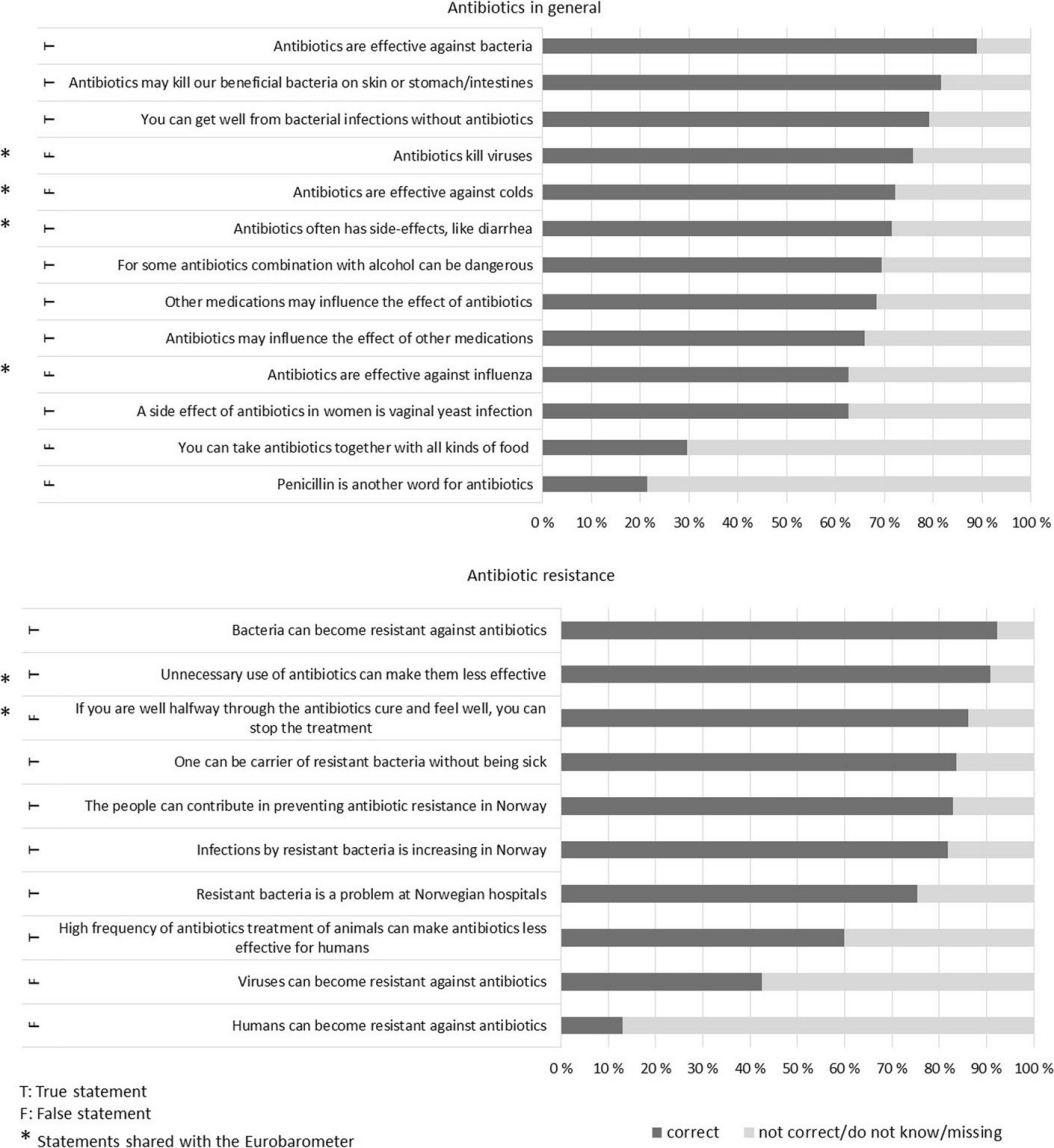
Proportion of participants with correct identification of true and false statements on antibiotics and antibiotic resistance

Figure 1 shows the proportion of participants with appropriate response to each of the 13 general and 10 resistance specific statements on antibiotics. The knowledge level was generally high for most statements. Satisfactory knowledge per statement (> 80% of the participants gave correct answer) was achieved for two out of 13 statements on antibiotics in general and for six out of ten statements on antibiotic resistance. More than 90% knew that bacteria can become resistant against antibiotics and that unnecessary use of antibiotics can make them less effective. Items with particularly low knowledge (< 50% of the participants gave appropriate response) included “you can take antibiotics with all kinds of food”, “penicillin is another word for antibiotic”, “viruses can become resistant against antibiotics” and “humans can become resistant against antibiotics”.

The mean overall antibiotic knowledge score was 15.6 (67.8% of maximum score), relatively higher on antibiotic resistance compared with general knowledge (Table 2). The corresponding estimated average knowledge score for the general population was 14.8, weighted for age, gender and education level.

**Table 2:** Scores on antibiotic knowledge, general beliefs about medicines (BMQ) and attitude toward antibiotic use among Norwegian pharmacy customers

Knowledge of antibiotics (score range)	Mean score (SD)		Percent of max score	
AB general knowledge (1–13)	8.5	(2.8)	65.4%	
AB resistance knowledge (1–10)	7.1	(2.1)	71.0%	
Total AB knowledge (1–23)	15.6	(4.4)	67.8%	
Beliefs about medicines^a^ (score range)	Mean score (SD)		Mean score/4^b^ (SD)	
BMQ benefit (4–20)	16.2	(2.4)	4.1	(0.6)
BMQ harm (4–20)	10.3	(2.7)	2.6	(0.7)
BMQ overuse (4–20)	12.7	(2.4)	3.2	(0.6)
Attitude toward antibiotic use^a^ (score range)	Mean score (SD)		Mean score/2^b^ (SD)	
Restrictive attitude supported (2–10)	9.2	(1.1)	4.6	(0.6)
Non-restrictive attitude supported (2–10)	5.2	(1.9)	2.6	(1.0)

^a^Five-point Likert scale per statement (from strongly disagree = 1 to strongly agree = 5), 4 statements per BMQ domain, 2 statements per attitude domain

^b^Divided by the number of statements per domain, i.e. Likert scale 1–5 per domain

### General beliefs about medicines and attitudes toward antibiotic use

The BMQ scores show that the study population had an overall positive view of the value of medications with a mean per subdomain score of 4.1 for benefit, 2.6 for harm and 3.2 for overuse (Table 2). The study population showed a restrictive attitude toward antibiotic use with particularly high score on the restrictive items, and low score on less restrictive items (Table 2).

### Factors associated with antibiotic knowledge

Results from the multiple linear regression analysis (Table 3) showed that participants with a health professional background were more likely to have higher knowledge of antibiotics. Moreover, viewing medications in general as beneficial (high score on BMQ benefit) was associated with higher antibiotic knowledge. Knowledge scores according to aggregated BMQ categories (Table 1) show that for five out of six comparisons, the middle BMQ category (“uncertain”) had the lowest score, suggesting that the associations may not be linear. A less restrictive attitude toward antibiotic use was negatively associated with antibiotic knowledge, while a restrictive attitude was suggestive of higher knowledge of antibiotic resistance (borderline significance, *p* = 0.001).

**Table 3:** Association between various characteristics and knowledge of antibiotics and antibiotic resistance (multiple linear regression)

	Antibiotics, general knowledge^c^			Antibiotic resistance knowledge^c^		
	Coeff.	95% CI		Coeff.	95% CI	
Beliefs about medicines, BMQ score						
Benefit	**0.116**	**0.037**	**0.195**	**0.132**	**0.074**	**0.190**
Harm	−0.062	−0.141	0.018	−0.034	− 0.092	0.024
Overuse	0.075	−0.005	0.156	0.020	−0.039	0.080
Attitude antibiotics, score						
Restrictive	**0.165**	**0.006**	**0.324**	**0.190**	**0.073**	**0.307**
Less restrictive	**−0.161**	**−0.253**	**−0.069**	**− 0.190**	**−0.257**	**− 0.122**
Age^a^						
30–44 years	0.493	−0.147	1.133	**0.923**	**0.452**	**1.394**
45–59 years	0.719	0.092	1.345	**1.092**	**0.631**	**1.553**
> =60 years	0.497	−0.258	1.252	**1.256**	**0.701**	**1.812**
Gender^a^						
Men	**− 0.867**	**− 1.253**	**−0.481**	0.151	−0.134	0.435
Education^a^						
Upper secondary school	0.188	−0.504	0.880	0.211	−0.298	0.720
College/University ≤3y	0.219	−0.506	0.944	0.602	0.069	1.136
College/University >3y	0.808	0.063	1.553	**0.977**	**0.429**	**1.525**
Health professional^b^						
Yes	**1.670**	**1.264**	**2.076**	**0.905**	**0.606**	**1.203**
Do not know	1.412	0.156	2.668	−0.259	−1.183	0.664
Antibiotics use last 12 months^b^						
Yes	0.333	−0.056	0.721	0.184	−0.101	0.470
Do not remember	−1.026	−2.458	0.406	−1.451	−2.504	−0.397
Healthy						
Score	0.081	−0.117	0.278	0.064	−0.082	0.209
Chronic disease^b^						
Yes	0.550	0.112	0.988	0.009	−0.313	0.331
Will not tell	−0.065	−1.307	1.177	−0.147	−1.061	0.767
Medication^b^						
Some times	− 0.317	− 1.040	0.406	0.040	−0.492	0.572
Yes	−0.746	−1.217	−0.274	− 0.310	−0.657	0.037
Smoking^b^						
Some times	− 0.077	− 0.806	0.653	−0.329	− 0.866	0.208
Yes	0.071	−0.517	0.659	−0.077	−0.510	0.355
Marital status^a^						
Relationship	− 0.564	−1.274	0.146	0.251	−0.271	0.773
Single	−0.176	−0.589	0.236	0.006	−0.297	0.310
Other	−0.682	−1.779	0.414	−0.232	−1.038	0.575
Work^a^						
Not working	− 0.230	− 0.688	0.228	−0.469	− 0.805	−0.132
Student	−0.113	−0.859	0.633	−0.065	− 0.614	0.484
Retired	−0.831	−1.464	−0.198	− 0.450	−0.915	0.016

*CI* Confidence Interval*, Coeff*. beta coefficient from univariate linear regression

The numbers (coefficients and accompanying confidence Intervals (CI)) represent change in knowledge score for antibiotics in general and antibiotic resistance respectively

Significant associations (*p* < 0.001) are marked in bold

^a^Reference category is “18–29 years” for age, “women” for gender, “primary/lower secondary school” for education, “married/cohabiting” for marital status and “active work” for work situation

^b^Reference category is “no”

^c^Ten participants had “do not know” on all 23 knowledge items while 59 had missing on at least one item. Number of missing per knowledge item varied from 0 to 11. All missing values were set to zero

Knowledge of antibiotics and antibiotic resistance differed according to some of the background characteristics. Men were more likely to have lower general knowledge of antibiotics than women, but there were no gender difference in knowledge of antibiotic resistance. Higher age and higher education was associated with higher knowledge of antibiotic resistance but not of antibiotics in general. For education, this might have been a question of statistical power, as the trend in the coefficients were similar for both scores. The knowledge scores according to age category (Table 1) suggested an inverse u-shaped rather than linear relationship between age and general knowledge, with lower knowledge among the youngest and oldest participants.

According to the univariate regression (see Additional file 1), viewing medicines as harmful (high score on BMQ harm) and not being in active work was negatively associated with antibiotic knowledge, but this was no longer statistically significant in the multivariable analysis. The remaining sociodemographic factors showed no significant impact on antibiotic knowledge. Neither did any of the health related factors.

## Discussion

This study among Norwegian pharmacy customers shows a relatively high public knowledge of antibiotics and antibiotic resistance comparable to studies from Sweden [21, 22], and higher than in many other countries [23]. The higher knowledge scores on antibiotic resistance compared with general knowledge of antibiotics may be due to the increasing focus on antibiotic resistance in media in later years [17]. This could also be seen as a result of the national strategy against antibiotic resistance [7], including the national information campaign [13], which may have spurred the media focus.

Similarly to previous studies [21, 22, 27], we identified that people tend to know that antibiotic resistance is a result of inappropriate or unnecessary antibiotic use, and that it makes the antibiotics less effective, but they do not necessarily know what antibiotic resistance or unnecessary use is. For instance, our data show that almost 90% believe that humans can become resistant against antibiotics and many do not differentiate between bacteria and virus, with over 30% saying that antibiotics are effective against viruses, colds or influenza. Some may not know that colds and influenza are all viral infections. Considering the six knowledge statements in common with the Eurobarometer [11], our study participants would have reached first place for knowing that antibiotics do not kill viruses (76%), but only tenth for correctly dismissing effectiveness against colds and influenza. To mitigate unnecessary use of antibiotics, it would be helpful if patients were aware of this. Our findings are in accordance with an interview study commissioned by the Norwegian Directorate of Health in 2016 (not published) [28]. Focus group interviews conducted among Norwegian parents showed that there is an interest in antibiotic resistance, but the concept is easily misunderstood.

The particularly low score on penicillin being another word for antibiotics is understandable as penicillin was the first marketed antibiotic and a tremendously important medicinal discovery. It is also the most used antibiotic in Norway and the name has become fixed in people’s minds. Although this may seem trivial, it adds unwanted confusion. For instance, patients may say they are “allergic to penicillin”, and so the doctor unnecessarily prescribes a broad-spectrum antibiotic. The statement that antibiotics can be taken together with food may be somewhat ambiguous, as many antibiotics are not noticeably influenced by food intake, thereby leading to uncertainty among the participants. Even for oral penicillin, where food intake inhibits the absorption, avoiding food is less important for optimal effect than administering the doses at regular, frequent time intervals [29].

Antibiotic knowledge was positively associated with positive beliefs about medications and with increasingly restrictive attitude toward antibiotic use. Our results are in line with previous reports showing that beliefs and attitudes are important for knowledge of medications [30], including antibiotics [20, 22, 31]. Interventions aiming to increase knowledge of antibiotics may promote restrictive attitude toward antibiotics, thereby contributing to reduced utilization and consequently lower selective pressure and less antibiotic resistance. A similar influence on beliefs about medications may contribute to stronger adherence, and thereby optimized effect of the antibiotic whenever a prescription is justified. This has been shown in studies of selected patient groups [32, 33].

Being male, having low education and particularly not having a health professional background was associated with a lower level of knowledge of antibiotics. The gender difference may reflect that women generally are more concerned with health issues, for instance through taking on a greater responsibility for children’s or parents’ health [34], or specifically through experience with antibiotic treatment. The prevalence of antibiotic use in Norway is approximately 50% higher among women than men [35], and the difference is largest among young adults [36]. Regarding our questionnaire, we should also remember that awareness of vaginal yeast infection probably is higher among women and may contribute to the identified gender difference in general knowledge. Age seems influential as younger people show lower knowledge. This may indicate that the knowledge of antibiotic resistance comes from higher education or experience with antibiotic use, and is not acquired in the basic compulsory educational system. Also, healthy young people are probably less inclined to show interest in disease related topics presented in traditional media. Our results on gender and age influence are in accordance with unpublished results from a web survey commissioned by the Norwegian Directorate of Health in 2016 [26]. Also noteworthy, is that the national campaign, which was ongoing during our data collection period, was targeted toward mothers of small children and adults 25–44 years [14].

As shown by a recent systematic review, the effect of public information campaigns on antibiotic prescribing tends to vary between different populations and study designs [12]. A Norwegian study showed a reduced frequency of bacterial infections presented at emergency centres during autumn 2009 compared with previous years, suggesting an impact of the public hygiene campaign during the H1N1 influenza pandemic in 2009 [37]. According to our findings, a campaign to increase the level of public knowledge of antibiotics in Norway would probably be most effective if targeted toward people in the less academic and particularly the male dominated professions or occupations. Additionally, increased focus on antibiotics at the primary and secondary school level would increase the knowledge among the young generations and minimize knowledge gaps due to education level when they grow older. At present, neither the core curriculum for primary, secondary and adult education in Norway nor the natural science subject curriculum specify learning objectives regarding antibiotics [38]. A general aim is “*to enable pupils to explain how the body protects itself against illness and how one can prevent and treat infectious disease”*, which leaves the degree of depth on antibiotics to the teachers. A new national curriculum will be developed during 2018 with implementation planned from 2019. This represents a timely opportunity to increase the focus and knowledge of antibiotic resistance among future generations. The curriculum should perhaps make room for “…*one of the biggest threats to global health, food security, and development”* [1].

As the greatest knowledge gaps seems to be in general antibiotic knowledge, a campaign should focus on this area. Pharmacies are excellent arenas to spread correct information on antibiotics. Particularly relevant issues would be the three items with lowest rate of correct answers within antibiotics in general: side effects of antibiotics, combination with food and the concept of antibiotics versus penicillin. However, as pharmacies intercept not only people with antibiotic prescriptions but also buyers of non-prescription cough and cold products, there would be frequent opportunities to explain the difference between bacterial and viral infections, which would support the comprehension of antibiotic resistance.

Given that the national campaign was effective, which might have been the case as our findings show that women and the middle age group have higher scores, we think the Norwegian Directorate of Health should commission a similar campaign targeted toward men in vocational professions and fathers in general. This might imply different means of action, for instance different case examples and targeted messages in the campaign material. A reasonable aim for a national campaign would be to increase the public knowledge to above 80% for all knowledge items in our study.

### Strengths and limitations

To our knowledge, this is the first study of its kind in Norway, thereby providing useful information on a highly relevant topic. Although our study sample in some respects deviates from the general population, the highly significant associations with antibiotic knowledge that we have identified from our material, would in all likelihood also be significant in the general population.

The master students were present to answer questions from the participants at all times during data collection. This has contributed to the completeness of data, with the maximum proportion of missing per question/statement being 2.2% (work situation). Combined with imputation of missing values on BMQ and attitude score variables, this ensured that only 6.9% of the population were excluded in the multiple linear regression analysis. The facilitation included help for participants who had difficulties understanding some of the questions. This implies a potential for overestimation of knowledge if the students were too helpful, but also underestimation if help had not been given. The students were conscious not to influence the participants’ answering, so we assume low influence from the facilitation.

The cross-sectional design prevents us from drawing conclusions regarding causal relationships. For instance, high knowledge of antibiotics may just as likely be a prerequisite for restrictive attitudes toward antibiotics as vice versa.

The study population is not representative for the Norwegian general population ≥ 18 years. Our sample is somewhat older with 36% ≥60 years compared with 28% in the general population [39], women were overrepresented, and the proportion with higher education was 56% compared with 33% in the general population. The estimated population average antibiotic knowledge score weighted by age, gender and education is therefore understandably lower. The question is whether it is still an overestimate. According to Statistics Norway, 12% of the Norwegian population in 2016 had a health professional education [39]. The proportion was 27% in our study sample, and having a health professional background was the single factor with strongest influence on knowledge scores in our analysis.

There are no official statistics that demographically describe Norwegian pharmacy customers, i.e. our source population. Compared with the population who redeemed at least one prescription (The Norwegian Prescription Database, NorPD) in 2016, our study sample includes more women (68% versus 55% in NorPD), but the proportion ≥ 60 years is comparable (36% versus 37% in NorPD). However, the pharmacy customers in our study also include people merely buying over-the-counter medication and various products marketed for health and well-being. This may account for our higher proportion of younger people, and particularly women.

Our study sample is relatively large, but with a moderate response rate. The motivation to participate is probably higher among people with an interest in, or experience with, antibiotics, which may contribute to an overestimation of knowledge level. The proportion reporting antibiotic use during the previous 12 months is fairly high (30%) compared to the one-year prevalence from NorPD for 2016 (23%) [5], and comparable to the Eurobarometer (34%) [11]. However, our data do not consistently support that use of antibiotics during the previous 12 months is associated with antibiotic knowledge. The response rate was particularly low in Bergen (19%) compared with Tromsø (55%) and Skien (63%). However, we found no significant association between study site and antibiotic knowledge score (data not shown), suggesting that non-response bias is not a serious threat to our estimates and interpretations.

The knowledge and attitude statements in our questionnaire were not validated, but were mainly based on questionnaires used in previous studies [11, 26], and a face validity study was conducted to ensure comprehensibility. The interpretation of the results from the following three knowledge statements would have benefited from a more precise statement formulation: whether the antibiotics with food statement is true or false, whether penicillin is another word for antibiotics and whether humans can become resistant to antibiotics.

Lack of scientific studies measuring the public’s knowledge about antibiotics and antibiotic resistance is worrying given the global call to fight antibiotic resistance. This study contributes to fill this gap. Moreover, it adds to standard surveys, like the Eurobarometer, by including measures of beliefs about medicines, attitudes toward antibiotics as well as background characteristics, thereby addressing risk factors for low level of knowledge. The study reveals knowledge gaps that should be filled and groups that should be targeted for efforts to increase the antibiotic knowledge. However, a large-scale nation-wide study would be desirable.

## Conclusion

We have identified a high level of knowledge of antibiotics, particularly antibiotic resistance, among Norwegian pharmacy customers. This suggests that Norwegians are aware that antibiotic resistance poses a health threat, and that unnecessary use of antibiotics is driving this threat. Nevertheless, there seems to be a knowledge gap when it comes to understanding the rationale behind the resistance problem. We suggest that action is taken to increase public knowledge of antibiotics, particularly among people without a health professional background, in less academic (vocational) and/or male dominated occupations. We also suggest that fathers, as well as mothers, are targeted in campaigns directed towards families with young children. Additionally, the upcoming revision of the national curriculum for primary and secondary school should include learning objectives regarding antibiotics and antibiotic resistance.

## Additional file

Additional file 1: Results from univariate linear regression analyses (PDF 506 kb)

## Abbreviations

BMQ: Beliefs about medicines questionnaire
DDD: Defined Daily Dose
EU: European Union
MRSA: Methicillin Resistant *Staphylococcus aureus*
NorPD: The Norwegian Prescription Database
SD: Standard deviation
VRE: Vancomycin-resistant enterococci
WHO: The World Health Organization
