# Supplementary material for: Knowledge of antibiotics and antibiotic resistance among Norwegian pharmacy customers – a cross-sectional study
Source: BMC Public Health. 2019 Jan 15;19:66. doi: 10.1186/s12889-019-6409-x (PMC6332570; doi:10.1186/s12889-019-6409-x)
Supplement: Supplementary file 1 — Results from univariate linear regression analyses (PDF 506 kb) [file 12889_2019_6409_MOESM1_ESM.pdf]

Supplementary table 1 Association between various characteristics and knowledge of antibiotics and antibiotic resistance (univariate linear regression)

	Antibiotics, general			Antibiotic resistance		
	Coeff.	95% CI		Coeff.	95% CI	
Beliefs about medicines, BMQ						
Benefit	**0.163**	**0.086**	**0.241**	**0.188**	**0.132**	**0.243**
Harm	**-0.183**	**-0.252**	**-0.115**	**-0.146**	**-0.196**	**-0.096**
Overuse	-0.002	-0.078	0.073	-0.037	-0.092	0.018
Attitude antibiotics, score						
Restrictive	**0.333**	**0.170**	**0.496**	**0.316**	**0.198**	**0.434**
Less restrictive	**-0.239**	**-0.334**	**-0.144**	**-0.230**	**-0.298**	**-0.162**
Age*						
30-44 years	0.784	0.198	1.369	**0.929**	**0.501**	**1.357**
45-59 years	0.727	0.177	1.277	**0.906**	**0.504**	**1.309**
>=60 years	-0.472	-0.981	0.036	0.477	0.105	0.849
Gender*						
Men	**-1.312**	**-1.704**	**-0.910**	-0.055	-0.347	0.238
Education*						
Upper secondary school	0.657	-0.061	1.37	0.411	-0.101	0.922
College/University ≤3y	0.949	0.213	1.685	**1.151**	**0.626**	**1.675**
College/University >3y	**1.807**	**1.072**	**2.541**	**1.658**	**1.135**	**2.181**
Health professional**						
Yes	**2.147**	**1.748**	**2.547**	**1.057**	**0.755**	**1.359**
Do not know	1.159	-0.115	2.433	-0.448	-1.413	0.516
Antibiotics use last 12						
Yes	**0.434**	**0.027**	**0.841**	0.123	-0.174	0.419
Do not remember	-1.474	-3.077	0.129	-1.484	-2.652	-0.317
Healthy						
score	-0.003	-0.201	0.173	-0.067	-0.203	0.069
Chronic disease**						
Yes	0.346	-0.034	0.726	-0.004	-0.282	0.274
Will not tell	-0.180	-1.505	1.145	-0.427	-1.395	0.541
Medication**						
Some times	-0.058	-0.823	0.708	-0.333	-0.891	0.225
Yes	**-0.462**	**-0.859**	**-0.065**	-0.204	-0.493	0.086
Smoking**						
Some times	-0.077	-0.870	0.717	-0.588	-1.164	-0.013
Yes	-0.141	-0.759	0.477	-0.376	-0.824	0.071
Marital status*						
Relationship	-0.874	-1.569	-0.180	-0.323	-0.828	0.183
Single	-0.483	-0.914	-0.051	-0.415	-0.729	-0.101
Other	-0.894	-2.065	0.278	-0.716	-1.569	0.137
Work*						
Not working	-0.236	-0.715	0.243	**-0.670**	**-1.023**	**-0.317**
Student	-0.786	-1.438	-0.133	**-0.963**	**-1.444**	**-0.483**
Retired	**-1.531**	**-1.998**	**-1.065**	**-0.746**	**-1.090**	**-0.403**

CI = Confidence Interval, Coeff.= beta coefficient from univariate linear regression
The numbers (coefficients and accompanying confidence Intervals (CI)) represent change in knowledge score for antibiotics in general and antibiotic resistance respectively.
Significant associations (p<0.001), are marked in bold.
* Reference category is “18-29 years” for age, “women” for gender, “primary/lower secondary school” for education, “married/cohabiting” for marital status and “active work” for work situation.
** Reference category is “no”.
*** Ten participants had “do not know” on all 23 knowledge items while 59 had missing on at least one item. Number of missing per knowledge item varied from 0 to 11. All missing values were set to zero.
